# Cryoprotective Response as Part of the Adaptive Strategy of the Red Palm Weevil, *Rhynchophorus ferrugineus*, against Low Temperatures

**DOI:** 10.3390/insects13020134

**Published:** 2022-01-27

**Authors:** Trinidad León-Quinto, Arturo Serna

**Affiliations:** 1 Área de Zoología, Departamento de Agroquímica y Medio Ambiente, Universidad Miguel Hernández, E3202 Elche, ALC, Spain; 2 Instituto de Bioingeniería, Universidad Miguel Hernández, E3202 Elche, ALC, Spain; 3 Departamento de Física Aplicada, Universidad Miguel Hernández, E3202 Elche, ALC, Spain; arturo.serna@umh.es

**Keywords:** red palm weevil (RPW), palm pest, tropical insect, coleoptera, cold hardening, adaptive strategy, low molecular weight substances, cryoprotectant, glucose, glycerol

## Abstract

**Simple Summary:**

Low environmental temperature acts as a barrier that imposes limits on the geographic distribution of insects. However, due to Earth’s global warming, temperature might no longer be an impediment for insects to colonize some new areas. The spread of pest insects will depend on their adaptive response to cold periods and to thermal anomalies associated with climate change. In this study we analyzed whether the red palm weevil (RPW), one of the worst palm pests worldwide and native to warm areas, has physiological mechanisms that could configure an adaptive response to cold. We find that RPW is capable of rapidly producing substances that reduce chill injuries, primarily glucose as well as glycerol and several amino acids (mainly alanine). Therefore, this work shows for the first time that RPW is able to develop adaptive biochemical responses to deal with low temperatures, similar to those used by overwintering insects. Our results could be useful to improve models predicting the possible spread of RPW to new geographical areas, and also to try to prevent its adaptive response by disrupting the metabolic pathways regulating the involved substances.

**Abstract:**

The red palm weevil (RPW), *Rhynchophorus ferrugineus*, is one of the worst palm pests worldwide. In this work, we studied the physiological basis underlying its adaptive strategy against low temperatures. Specifically, we analyzed the main low-molecular-weight biochemical substances acting as possible endogenous cryoprotectants, as well as their efficiency in reducing cold injury by preserving K^+^/Na^+^ homeostasis. Wild pre-pupae were cold-treated (5.0 ± 0.5 °C) or non-treated (23 ± 1 °C) for 7 days. We then determined the levels of: (a) glucose, trehalose and glycerol, spectrophotometrically, (b) amino acids, by liquid chromatography and (c) potassium and sodium, by inductively coupled plasma mass-spectrometry. Cold-treated larvae increased their potassium level, suggesting some degree of chill injury. However, part of the cold-exposed animals was able to develop an efficient overall cryoprotective response which primarily includes glucose, as well as glycerol and several amino acids (mainly alanine). Our study shows for the first time that RPW is capable of deploying effective physiological mechanisms for a rapid response to cold, which could be relevant to improving predictive models of geographic distribution, especially in a context of climate change. The knowledge of the specific molecules involved would allow future studies to try to prevent its adaptive strategy, either by natural or chemical methods.

## 1. Introduction

The red palm weevil (RPW), *Rhynchophorus ferrugineus* Olivier (Coleoptera: Dryophthoridae), is considered to be one of the most destructive palm pests in warm areas worldwide. It is native to Southeast Asia and Oceania. However, due to the movement of live infested palms, RPW has widely spread to other regions of Asia, the Middle East, the Mediterranean Basin and Caribbean [1], giving rise to huge environmental and economic losses. Recent reports [2] indicate that palm trees worth up to 483 million euros have been destroyed or infested in the Mediterranean region, primarily by RPW. In Spain, the cost caused by RPW in 2005–2009 was about 44.5 million euros, particularly in the autonomous community of Valencia (eastern Spain) with a cost during this period of about 27 million euros [3]. In this latter region, the pest has also affected some areas of high cultural value such as the “Palmeral de Elche”, a palm grove declared a World Heritage Site by UNESCO.

Detection of RPW is difficult because it spends most of its life cycle inside the palm tree, where larvae make tunnels and feed on its inner contents. Only mature grubs go to the periphery of the stem, where they prepare a cocoon made of palm fibers before entering the pupal stage [3]. Huge efforts have been made to develop and implement different control methods to manage this pest, including survey and removal of heavily infested trees, chemical control (see [4] for a review) and different trapping systems [5,6], as well as biological control using entomopathogenic nematodes [7] and fungi [8]. The efficiency of some of these methods can be improved by means of population dynamics models that help to decide the optimal moment for their application [9]. In the same way, identifying the possible spread of RPW using geographic distribution models [10,11] can help to focus effective inspection and quarantine protocols.

The geographic distribution of insects is strongly correlated with their ability to tolerate abiotic variability in the environment [12] and, in particular, with their tolerance to low temperatures [13]. Indeed, the low environmental temperature disrupts the homeostasis of insects [14], which puts their survival at risk and acts as a barrier that imposes geographic limits on their distribution. In recent decades, human activity and climate change have had a considerable impact on the distribution of all species, including insects [15,16]. The redistribution of species both on a regional and global scale in response to global warming [17] is now considered a global change driver on its own, with far-reaching implications for ecosystems and human health [18]. As cold areas are turning into warmer ones due to climate change [19], temperature might no longer be an impediment for insects to colonize these new geographical locations. In particular, RPW has demonstrated invasion capacity even in areas with unfavorable winter temperatures. In some of these areas, the possible adverse effects of the external air temperature may be moderated within the protective confines of the palm trunk by the heating associated with microbial activity (reviewed in [4]). Making predictions that accurately capture how species will respond to temperature variations linked to climate change requires knowledge of the cold adaptability of insects and their low-temperature biology.

Insects have developed a diversity of responses to survive cold stress, such as morphological, behavioral, ecological, physiological and biochemical adaptations [20]. Each species adopts a different combination of these survival responses and, therefore, requires a specific study [21]. The mechanisms underlying the cold tolerance of insects have been extensively studied for species native to cold areas (e.g., [22,23,24,25]), but remain poorly understood for species inhabiting warm geographic areas. In overwintering insects, part of the adaptive response is generally based on the accumulation over long periods of low molecular weight substances [24,25,26,27,28]. Among these substances, sugars (e.g., trehalose and glucose), polyols (e.g., glycerol) and free amino acids (e. g., proline) are well-known cryoprotectants that contribute to stabilize cell membranes [24], prevent the loss of ion homeostasis [28] and reduce the probability of ice formation by lowering the hemolymph freezing point [29,30]. However, to survive sudden low-temperature events, insects need much faster mechanisms than those associated with overwintering. Studies reported for some species, both native to cold and warm areas, indicate that rapid adaptation seems to be based on the production of the same type of cryoprotectants as in seasonal cold hardening [30], probably sometimes in combination with the synthesis of heat-shock proteins [31] or other mechanisms [32].

Previous studies [33,34] have shown that RPW prepupal and pupal stages have a remarkable cold tolerance, with a lethal temperature as low as 0 °C. In addition, in a recent study [35] we showed by computed tomography and holographic microscopy that the RPW larval–pupal transition continues almost undisturbed even during the quiescence induced by cold stress. Indeed, wild pre-pupae subjected to low temperatures did not stop their development, and only showed slight developmental delays at the level of hemolymph content and the integumental roughness.

The aim of this work is to study the physiological basis underlying the adaptive strategy of RPW to deal with cold. Our hypothesis is that RPW has physiological mechanisms that configure an adaptive response against low environmental temperatures. These mechanisms would be based on the mobilization and increase of different substances, in a similar way to how overwintering insects do. To this end, we will analyze for the first time the main biochemical substances acting as possible endogenous cryoprotectants. In particular, we will measure the concentration in hemolymph of glucose, trehalose, glycerol and a wide range of free amino acids. The levels of these metabolites in RPW larvae subjected to cold stress will be then compared with those obtained in larvae that remained at room temperature. In addition, we will test the potential cryoprotective effect of these substances through the levels in the hemolymph of potassium and sodium, one of the indicators commonly used as a measure of chill injury [36,37,38]. Indeed, low temperature in insects impairs the activity of ion pumps, causing sodium leak away from the hemolymph and a progressive increase in the hemolymph potassium concentration. This hyperkalemia, combined with the depolarizing effects of low temperatures, causes a cascade of injuries that could become irreversible and eventually lead to death. Therefore, species with efficient cold-hardening mechanisms generally show relatively stable sodium and potassium levels when subjected to cold stress [28,39]. 

Understanding and predicting how RPW is capable of displaying a physiological response to deal with low temperatures is important to deepen the knowledge of cold adaptation in insects from warm areas. At the same time, it could be relevant to improve models of insect distribution and hence to successfully manage the possible spread of this pest towards new geographical areas, especially in a context of global warming. In addition, knowledge of the specific molecules involved in the RPW cold-hardiness could also be useful in trying to prevent this adaptive response by disrupting the metabolic pathways regulating these substances.

## 2. Materials and Methods

### 2.1. Insect Sampling

Wild RPW larvae were collected in spring and autumn 2019–2021 from infested palm trees, *Phoenix canariensis* and *Phoenix dactylifera*, located in the palm grove of Elche, Spain. Larval removal was carried out by qualified staff from TRAGSA, the public company that carries out the conservation of this palm grove. After collection, larvae were immediately taken to the laboratory, where we selected those in a wandering or prepupal stage, which is the larval stage most exposed in wild conditions to the thermal characteristics of the environment [3]. We then divided the selected animals into two groups:

(a) Cold-treated group: formed by individuals placed for seven days in a climatic chamber at low-temperature conditions (5.0 ± 0.5 °C). These individuals entered into a state of quiescence, where they remained until they returned to warm temperatures.

(b) Non-treated group: formed by individuals that remained for seven days in a room under controlled ambient temperature (23 ± 1 °C). This temperature corresponds to the average found in autumn and spring for the periphery of the palm tree stems in the southeast of Spain [3]. Individuals in this group generally had a behavior of feeding cessation, very likely due to their wandering prepupal stage. In order to homogenize the conditions of this group, all its individuals were subjected to starvation. Since cold-treated individuals also starved due to their quiescent state, the environmental temperature was the main external factor that distinguishes the seven-day development of both groups.

In order to quantify all measured metabolites, we extracted hemolymph from both groups of larvae. Non-treated animals were anesthetized for 1 min at −20 °C to immobilize them, while cold treated pre-pupae did not require immobilization because they were in quiescence. Larvae integument was pierced by a needle and hemolymph of each individual was collected into 1.5 mL Eppendorf tubes, centrifuged and stored at –80 °C until use.

### 2.2. Measurement of Free Glucose, Trehalose and Glycerol in Hemolymph

Glucose and trehalose levels were determined spectrophotometrically using assay kits from Merck/Sigma-Aldrich, Darmstadt, Germany (MAK263) and Megazyme, Bray, Ireland (K-TREH), respectively. For each type of quantification, hemolymph samples were taken from 15 non-treated and 16 cold-treated individuals, and then prepared following the manufacturer’s instructions. Absorbance was measured (two replicates per individual) at 570 nm and 340 nm for glucose and trehalose quantification, respectively.

The amount of free glycerol was quantified by enzymatic determination incubating the samples with 0.8 mL free glycerol reagent (F6428 Merck/Sigma-Aldrich) at room temperature for 15 min. We used hemolymph samples taken from 14 non-treated and 15 cold-treated individuals. After incubation, glycerol concentration was spectrophotometrically quantified from the absorbance at 540 nm of each sample and that of a pure glycerol standard (G7793, Merck/Sigma-Aldrich). Procedure and calculation of the glycerol content was performed following the manufacturer’s instructions.

### 2.3. Measurement of Free Amino Acids, Potassium and Sodium in Hemolymph

To carry out the quantification of amino acids, potassium and sodium, 14 samples per group (cold treated and non-treated) were used. Each sample consisted of a pool (1 mL) of the hemolymph collected from 3–5 individuals.

Free amino acids were determined by liquid chromatography coupled by triple quadrupole mass spectrometry (LC-TQ system, Evoq-Elite Bruker, Germany). From each sample, 100 µL were taken, diluted in Milli-Q water up to 100 mL and filtered with 0.45 µm nylon filters. A final volume of 5 µL was then injected into the LC-TQ system using a Phenomenex Luna NH2 column (150 mm × 2.1 mm, 3 µm).

Potassium and sodium hemolymph levels were determined by inductively coupled plasma mass spectrometry (ICP-MS). We took 100 µL from each sample, which were diluted in Milli-Q water up to 50 mL. An aliquot (5 mL) was continuously aspirated by a peristaltic pump and a final volume of 1 mL was then introduced into the ICP-MS system (Agilent 7700e, Santa Clara, CA, USA).

### 2.4. Statistical Analysis 

Statistical analyses were performed with the XLStat software package (Addinsoft, New York, NY, USA). 

In most cases, we had a single factor (the concentration of a certain substance) and two treatments (non-treated and cold-treated larvae). For each substance, we compared the *k* = 2 means (one for each treatment) by applying the one-way ANOVA test (equivalent for *k* = 2 to Student’s *t*-test). Since this test requires a normal distribution, we also applied the Shapiro–Wilk normality test. Only for one amino acid, valine, this test rejected the normality hypothesis. In this case, in addition to the ANOVA test, we also applied the non-parametric Mann–Whitney test, which does not require the assumption of normality. In such a case, the one-way ANOVA and Mann–Whitney tests led to the same conclusion.

Within our study of sodium and potassium levels, we also compared the means of *k* = 3 treatments (non-treated and two subpopulations of cold-treated larvae). In this case, after the ANOVA test, we applied the LSD post-hoc test to identify the statistically different treatments.

In order to detect statistical differences, a *p* < 0.05 level of significance was applied in all the analyses.

## 3. Results

### 3.1. Glucose and Trehalose

**Figure 1:**
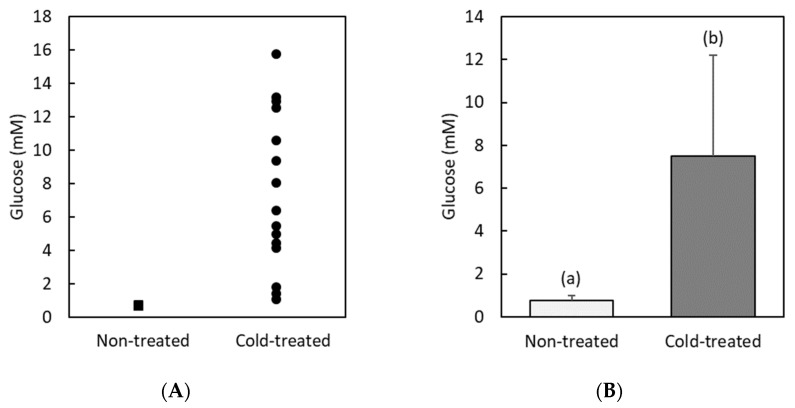
Hemolymph glucose concentration (mM) in non-treated and cold-treated groups of red palm weevil pre-pupae larvae: (**A**), individual values (*n* = 15 non-treated and *n =* 16 cold-treated larvae); (**B**) average values. Error bars indicate standard deviation. Labeling with different letters indicates that concentrations are significantly different (in this case, *F* = 30.5, *df* = 30, *p* < 0.001).

Figure 1 shows the results concerning glucose levels in hemolymph. We see from this figure that non-treated animals have a low glucose concentration (average value: 0.8 ± 0.3 mM), with individual values that are always below 2 mM. Cold treated larvae present instead a ten-fold increase and a wide spread in their glucose level (average value: 8 ± 5 mM). In this latter group, there are individuals with a glucose concentration similar to that found for non-treated animals and others with a very high content of glucose in hemolymph. Statistical analysis clearly shows that the glucose levels of both groups are significantly different (*F* = 30.5, *df* = 30, *p* < 0.001).

**Figure 2:**
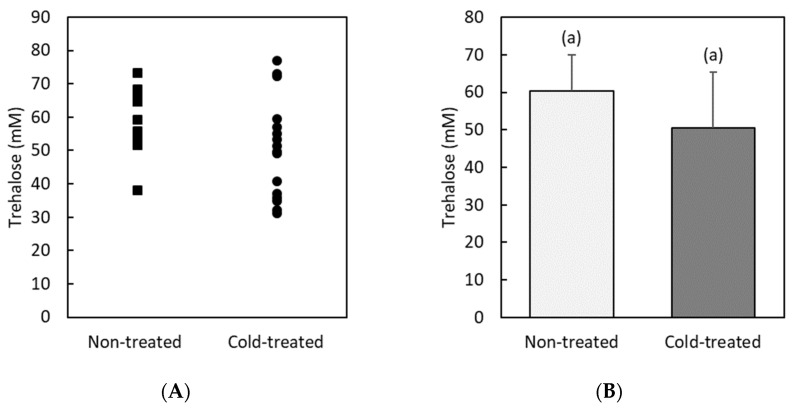
Hemolymph trehalose concentration (mM) in non-treated and cold-treated groups of red palm weevil pre-pupae larvae: (**A**) individual values (*n* = 15 non-treated and *n =* 16 cold-treated larvae); (**B**) average values. Error bars indicate standard deviation. Labeling with the same letters indicates that concentrations are not significantly different (in this case, *F* = 4.0, *df* = 30, *p* = 0.06).

On the other hand, we find that trehalose is a much more abundant sugar in hemolymph than glucose (Figure 2). The trehalose level is high and widely dispersed in both non-treated and cold-treated individuals. The average value of trehalose concentration in both groups (60 ± 10 mM and 50 ± 15 mM) does not present statistically significant differences (*F* = 4.0, *df* = 30, *p* = 0.06).

### 3.2. Glycerol

**Figure 3:**
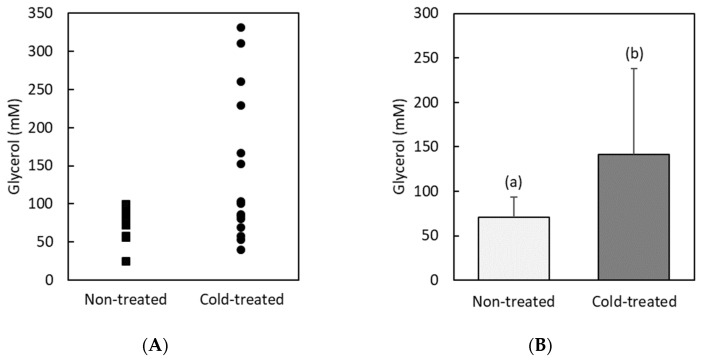
Hemolymph glycerol concentration (mM) in non-treated and cold-treated groups of red palm weevil pre-pupae larvae: (**A**) individual values (*n* = 14 non-treated and *n =* 15 cold-treated larvae); (**B**) average values. Error bars indicate standard deviation. Labeling with different letters indicates that concentrations are significantly different (in this case, *F* = 4.6, *df* = 28, *p* = 0.04).

Figure 3 shows the results concerning glycerol concentration in hemolymph. Similar to that previously found for glucose, we see that cold-treated animals have a high level of glycerol (141 ± 96 mM) as compared to that found for the non-treated group (71 ± 22 mM). Such a two-fold increase is statistically significant (*F* = 4.6, *df* = 28, *p* = 0.04). In addition, as in the case of glucose, we again find a wide spread in the cold-treated individual values of glycerol concentration, with animals having levels similar to those found for non-treated larvae (<125 mM) and others with a very high glycerol content.

### 3.3. Free Amino Acids

Our results for hemolymph amino acid concentration in non-treated and cold-treated red palm weevil pre-pupae are shown in Table 1. We find that the most abundant amino acids are proline, glutamine and lysine. However, the concentrations of these dominant amino acids in cold-treated and non-treated groups do not have significant differences. As a result, larvae subjected to a cold treatment do not present a significant change in the total concentration of amino acids.

Nevertheless, some amino acids show a modest but statistically significant increase in cold-treated samples. This is the case of alanine, serine and aspartic acid + asparagine, as well as five essential amino acids (histidine, threonine, leucine, isoleucine and valine). These amino acids show significative increments of 1.7-fold for histidine, 1.5-fold for alanine and 1.4-fold for serine, aspartic acid + asparagine, leucine, isoleucine, threonine and valine. None of the amino acids studied has a concentration with a significant decrease in cold-treated samples. The observed trend is always that the amino acid concentration increases with cold or remains unchanged.

### 3.4. Potassium and Sodium

**Figure 4:**
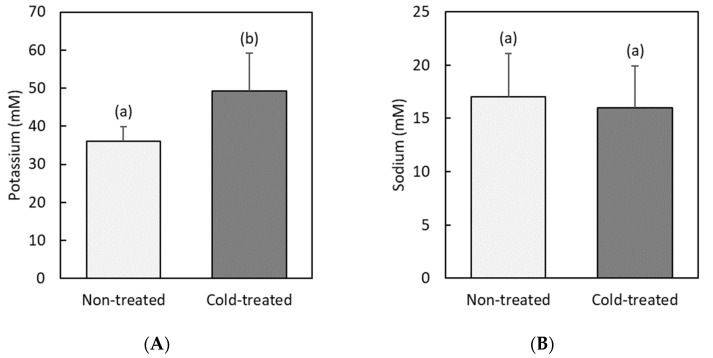
Concentrations (mM) of: (**A**) Potassium and (**B**) Sodium in hemolymph from non-treated and cold-treated groups. Error bars indicate standard deviations (*n* = 14 non-treated and *n =* 14 cold-treated samples, each sample containing the hemolymph of 3–5 individuals from the same group). Statistically different (*p* < 0.05) concentrations are labelled with different letters (*F* = 11.3, *df* = 27, *p* = 0.003 for potassium and *F* = 0.3, *df* = 27, *p* = 0.06 for sodium).

As remarked in the introduction, chill injury is closely associated with the loss of ion homeostasis and, in particular, with an increase in potassium ions and a decrease in sodium ones in the hemolymph. Therefore, in order to have some insight on the magnitude of chilling injury of cold-treated RPW larvae, we measured the hemolymph potassium and sodium levels. We see from Figure 4 that the sodium level in non-treated RPW larvae is low (17 ± 4 mM) and remains basically unchanged (16 ± 4 mM), with a slight non-significant decrease (*F* = 0.3, *df* = 27, *p* = 0.06) in cold-treated larvae. In contrast, we find that the potassium level in cold-treated animals is slightly but significantly (*F* = 11.3, *df* = 27, *p* = 0.003) higher (49 ± 9 mM) than in larvae that remained at room temperature (36 ± 4 mM).

Since glucose is the substance for which the highest increase (ten-fold) has been found after cold exposure, we have also tested a possible correlation between the accumulation of glucose, as a potential cryoprotectant, and the reduction of chill injury evaluated through potassium and sodium concentrations. To that end, we divided the cold-treated group into two subpopulations, according to their respective glucose accumulation against cold. In one of these subpopulations (cold-treated, LG), the samples come from larvae where low temperatures did not produce an increase in glucose content (<2 mM). In the other subpopulation (cold-treated, HG), the samples come from larvae that presented a high level of glucose (>2 mM) after being subjected to cold stress. We can expect that if glucose has acted as a cryoprotectant, potassium levels will be lower in the HG (high glucose) subgroup than in the LG (low glucose) subgroup.

**Figure 5:**
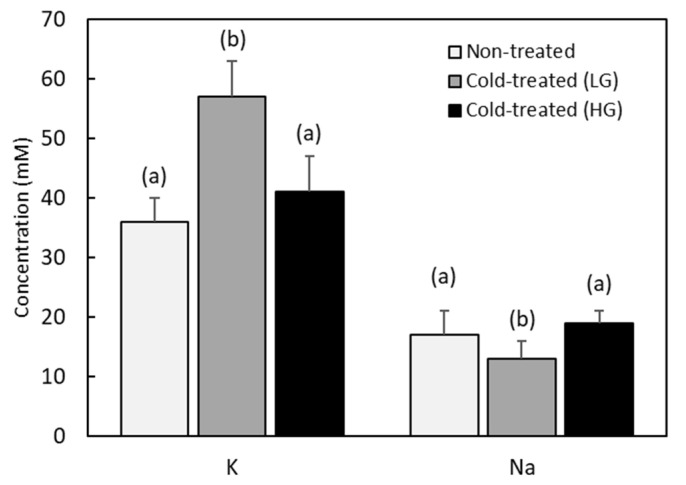
Hemolymph potassium and sodium levels in non-treated animals (*n* = 14) and in low-glucose (*n* = 7) and high glucose (*n* = 7) subgroups of cold-treated larvae. Error bars indicate standard deviations. Statistically different (*p* < 0.05) distributions are labelled with different letters (*F* = 28.7, *df* = 20, *p* < 0.001, *LSD* = 5.9 for potassium and *F* = 6.7, *df* = 20, *p* = 0.007, *LSD* = 3.1 for sodium).

As shown in Figure 5, we found that the LG and HG subpopulations have significantly different responses regarding ion balance (*F* = 28.7, *df* = 20, *p* < 0.001, *LSD* = 5.9 for potassium and *F* = 6.7, *df* = 20, *p* = 0.007, *LSD* = 3.1 for sodium). Indeed, the LG cold-treated subgroup has potassium levels significantly (means with differences >*LSD*) higher (57 ± 6 mM) than those observed in HG cold-treated (41 ± 6 mM) and non-treated larvae (36 ± 4 mM). At the same time, such a subpopulation presents a significantly (means with differences >*LSD*) lower sodium concentration (13 ± 3 mM) than those found in HG cold-treated (19 ± 2 mM) and non-treated larvae (17 ± 4 mM). However, the HG cold-treated subpopulation shows sodium and potassium concentrations that are not statistically different (means with differences <*LSD*) from those found in non-treated larvae.

## 4. Discussion 

In this work we have studied for the first time the possible cryoprotective response of the red palm weevil (RPW) as part of its adaptive strategy against low temperatures. In particular, we have measured the concentration in hemolymph of the main low-molecular-weight substances known as possible endogenous cryoprotectants in insects, mainly glycerol, sugars and free amino acids (see [24,40,41] for some reviews). As in previous studies with other species (e.g., [42,43,44]), only molecules whose levels significantly increased after cold stress have been considered as potentially cryoprotective substances. 

We find that glucose is by far the substance that increases the most in the hemolymph of cold-treated RPWs. Indeed, our results imply that cold-treated animals presented an average 10-fold increase in glucose concentration, compared to that found in non-treated individuals. This response had considerable variability, from individuals where the glucose level remained almost unchanged by cold stress to others with a very high glucose content. In contrast, although our results show that trehalose is the most abundant sugar in the RPW hemolymph, its level did not significantly change after cold stress. Similar results for glucose and trehalose have been found in the literature for the cryoprotective response of other insect species [1,32,45]. Indeed, a cold-induced 6 to 10-fold increase in glucose concentration has been previously reported for other tropical species [46] and some overwintering Coleoptera [47]. An increased concentration in hemolymph of glucose and other sugars contributes to stabilizing cell membranes [24] and preserving from cold-induced hyperkalemia [28].

The accumulation of polyols is another common finding for insect cold hardening [48]. They are involved in stabilizing protein structure and in reducing intracellular ice formation [24]. In this work, we have also found a significant 2-fold increase in the glycerol level of cold-treated RPW larvae. The spread of such changes was even wider than that of glucose, from individuals with almost unchanged levels to others where the hemolymph glycerol concentration increased to more than 300 mM. A similar (3-fold) increase in glycerol levels has previously been reported for some tropical beetle species [43]. Our results then suggest that glycerol is very likely another substance that could contribute to the cold adaptation response in RPW.

Less conclusive are our results regarding the concentration of free amino acids. In this paper, we have identified proline, glutamine and lysine as the most abundant amino acids in the RPW hemolymph, and found that their levels do not significantly change after cold stress. Consequently, the total amino acid concentration does not change either. Nevertheless, we have found that some other amino acids have a modest but significant increase in cold-treated larvae. Among these amino acids, the most abundant is alanine, which shows a 1.5-fold increase at low temperatures. Different authors [43,49,50,51] have reported high levels of alanine in insects subjected to cold stress, suggesting an important role of this amino acid in the stabilization of membranes and proteins [42,52,53]. However, it is not clear that alanine accumulation should always be interpreted as part of a cryoprotective response. Some authors [54] have instead pointed out that, in a number of cases, the increase in the alanine content could be due to other factors, such as the decrease in oxygen uptake resulting from a slower breathing rate at low temperatures. We also found modest but significant increases in the hemolymph of cold-treated animals of other non-essential amino acids, such a serine and aspartic acid + asparagine, as well as of five essential amino acids (histidine, leucine, isoleucine, threonine and valine). An increase in serine [54], aspartic acid [55] and essential amino acids [43] in overwintering larvae or cold-treated insects has been previously reported in the literature. However, as in the case of alanine, it is not clear whether the accumulation of these amino acids is part of a cryoprotective response or the result of different metabolic pathways triggered by cold. 

Having shown that levels of glucose and other substances increase after cold stress, we have measured one of the indicators of their cryoprotective effect. More specifically, we have tested whether the observed increase in these substances has been accompanied by a stability in sodium and potassium levels. Indeed, a common way of evaluating chill injury in insects is through their hemolymph potassium and sodium concentration [36,37,38]. An ineffective cryoprotective mechanism fails to preserve ionic homeostasis and therefore, cold induces an excess of potassium ions. This hyperkalemia, together with other depolarizing effects of low temperatures, causes a cascade of injuries that can finally lead to death. We have found that, as it is generally observed in chill-tolerant insects [56,57,58], RPW larvae have low basal sodium levels that remain stable after cold stress. However, we have also found a slight but significant increase in the potassium level of cold-treated larvae. This latter result suggests that on average the metabolic response of RPW has not been able to avoid some degree of chilling injury, probably due to the unequal response between cold-treated individuals, as observed through the wide scatter in the resulting cryoprotectant accumulation. We have then divided the cold-treated group into two subpopulations depending on their glucose concentration, the substance with the highest increase. We have found that individuals with low glucose levels after cold stress have significantly higher potassium and lower sodium concentrations than animals that remained at room temperature. On the contrary, the subpopulation with high glucose accumulation has potassium and sodium levels without significant differences with respect to those of non-treated larvae. This result reinforces the hypothesis that glucose has acted, most likely together with other substances (glycerol, alanine...), as a cryoprotectant that significantly reduces cold injury. 

## 5. Summary and Conclusions

The present study shows for the first time that RPW is able to develop a rapid non-seasonal cold hardening response, which includes the synthesis of low-molecular-weight biochemical substances. Among these substances, glucose has a crucial cryoprotective role, as well as glycerol and probably also some amino acids (mainly alanine). These results would provide the underlying physiological basis for previous studies showing that prepupae and pupae are the most resistant RPW stages to low temperatures, with a lethal threshold of 0 °C [33,34], and also for recent morphological studies [35] showing that the larval–pupal transition continues almost undisturbed during the quiescence induced by cold stress, with just a slight developmental delay. All these findings together, both the previous ones and those obtained in the present study, strongly suggest that RPW, a species native to warm regions, has a remarkable cold-tolerance.

Our results could be useful for future improvement of integrated pest management programs. On the one hand, the identification in RPW of effective physiological mechanisms for a rapid response to low temperature reinforces the need to improve predictive models of geographic distribution as a tool for the analysis of preventive and monitoring measures. These models include different climate scenarios, and there are attempts to incorporate cold-hardening details to infer the possible spread of this pest to regions with low winter temperatures [59]. Indeed, RPW is currently present in some regions with winter temperatures close to 0 °C, where heating by microbial fermentation inside infested palm trees facilitates the overwintering and non-stop development of this pest [60]. On the other hand, knowledge of the specific molecules used by RPW to deal with cold would allow future studies to try to prevent its adaptive response either by natural or chemical methods. For instance, it is known that imidacloprid alters glucose transporters in some animals [61] and causes a decrease in glucose levels in cockroaches [62]. The study of glucose or glycerol transporter blockers in RPW could be also another approach to be explored in future research.

## Figures and Tables

**Table 1:** Concentration of free amino acids in non-treated and cold-treated groups.

Amino Acid		Concentration (mg/mL)		ANOVA Values	
		Non-Treated	Cold-Treated	*F*	*p*
Aspartic acid + Asparagine	ASP	1.3 ± 0.3 ^a^	1.8 ± 0.3 ^b^	8.0	0.01
Glutamic acid + Glutamine	GLU	2.6 ± 0.5 ^a^	2.3 ± 0.4 ^a^	1.2	0.3
Alanine	ALA	1.8 ± 0.6 ^a^	2.7 ± 0.9 ^b^	4.9	0.04
Arginine	ARG	0.7 ± 0.2 ^a^	0.8 ± 0.2 ^a^	1.7	0.2
Cysteine	CYS	<0.2	< 0.2		
Phenylalanine	PHE	1.2 ± 0.3 ^a^	1.5 ± 0.2 ^a^	3.6	0.08
Glycine	GLY	1.6 ± 0.6 ^a^	1.6 ± 0.8 ^a^	0.01	0.9
Histidine	HIS	0.7 ± 0.2 ^a^	1.2 ± 0.2 ^b^	21.0	0.001
Isoleucine	ISO	1.1 ± 0.2 ^a^	1.5 ± 0.2 ^b^	13.2	0.003
Leucine	LEU	1.6 ± 0.3 ^a^	2.1 ± 0.4 ^b^	9.0	0.01
Lysine	LYS	2.1 ± 0.5 ^a^	2.6 ± 0.5 ^a^	3.6	0.08
Methionine	MET	<0.2	<0.2		
Proline	PRO	7.1 ± 2.2 ^a^	6.2 ± 1.4 ^a^	0.9	0.4
Serine	SER	0.8 ± 0.3 ^a^	1.1 ± 0.2 ^b^	5.6	0.04
Tyrosine	TYR	0.6 ± 0.2 ^a^	0.9 ± 0.2 ^a^	4.5	0.05
Threonine	THR	0.7 ± 0.2 ^a^	1.0 ± 0.1 ^b^	19.7	0.001
Tryptophan	TRY	<0.2	<0.2		
Valine	VAL	1.4 ± 0.2 ^a^	2.0 ± 0.2 ^b^	19.4	0.001
TOTAL	TOT	25 ± 3 ^a^	29 ± 4 ^a^	4.3	0.06

All values are mean ± sem (*n* = 14 non-treated and *n =* 14 cold-treated samples, each sample containing the hemolymph of 3–5 individuals from the same group). Values significantly different (*p* < 0.05) for the same amino acid are labelled with different letters. In the case of valine, the normal distribution was rejected (*p* = 0.03 in the Shapiro–Wilk test), so we also applied the non-parametric Mann–Whitney test, which led to the same conclusion of significantly different means (*U* = 3, *p* = 0.007).

## Data Availability

The data presented in this study are available in article.
